# Supplementary material for: Netcombin: An algorithm for constructing optimal phylogenetic network from rooted triplets
Source: PLoS One. 2020 Sep 18;15(9):e0227842. doi: 10.1371/journal.pone.0227842 (PMC7500971; doi:10.1371/journal.pone.0227842)
Supplement: S4 File — (ZIP) [file pone.0227842.s004.zip › SequenceData-Triplet/sequenceData-10-20-30-0-Triplet/length 300, numseq 30, rate 10^-7/7 seq.pdf]

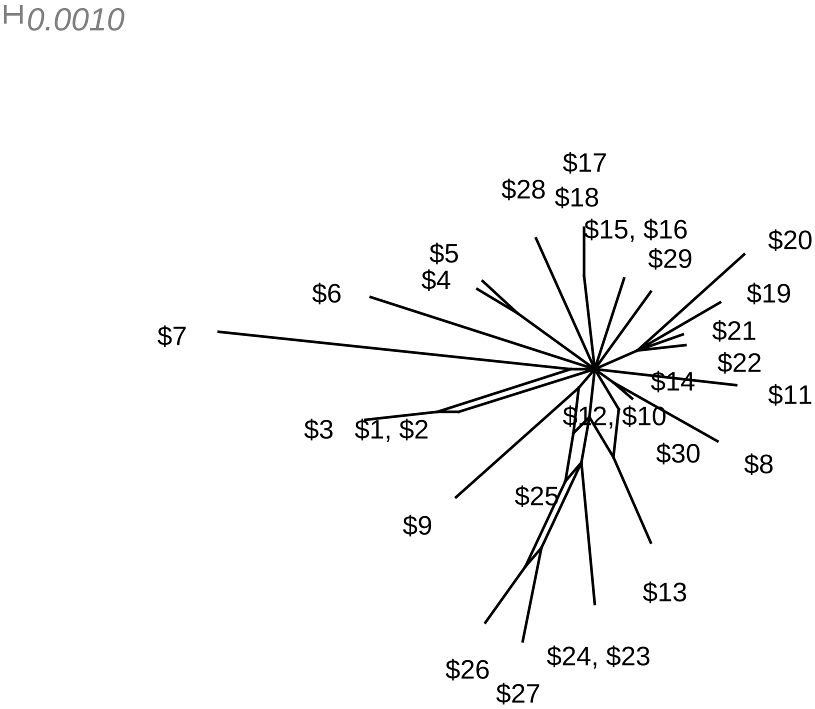
0.0010
$17
$28
$18
$15, $16
$20
$5
$29
$4
$6
$19
$7
$21
$22
$14
$11
$3
$1, $2
$12, $10
$30
$8
$25
$9
$13
$24, $23
$26
$27
